# Contributions of Motivation, Early Numeracy Skills, and Executive Functioning to Mathematical Performance. A Longitudinal Study

**DOI:** 10.3389/fpsyg.2017.02375

**Published:** 2018-01-15

**Authors:** Jessica Mercader, Ana Miranda, M. Jesús Presentación, Rebeca Siegenthaler, Jesús F. Rosel

**Affiliations:** ^1^Departamento de Psicología Evolutiva, Educativa, Social y Metodología, Universidad Jaume I, Castellón de la Plana, Spain; ^2^Departamento de Psicología Evolutiva y de la Educación, Universidad de València, València, Spain

**Keywords:** motivation, executive functioning, early numeracy skills, mathematical performance, longitudinal study

## Abstract

The main goal of this longitudinal study is to examine the power of different variables and its dynamic interactions in predicting mathematical performance. The model proposed in this study includes indicators of motivational constructs (learning motivation and attributions), executive functioning (inhibition and working memory), and early numeracy skills (logical operations, counting, and magnitude comparison abilities), assessed during kindergarten, and mathematical performance in the second year of Primary Education. The sample consisted of 180 subjects assessed in two moments (5–6 and 7–8 years old). The results showed an indirect effect of initial motivation on later mathematical performance. Executive functioning and early numeracy skills mediated the effect of motivation on later mathematic achievement. Practical implications of these findings for mathematics education are discussed.

## Introduction

Differences in the acquisition of skills in the initial learning of mathematics influence school and life success (Ancker and Kaufman, 2007; McCloskey, 2007; Geary, 2011). The first schooling years are fundamental to build and strengthen the base of knowledges and skills for the correct development of the different learning areas in the mathematical field. Early mathematical knowledge predicts later success in school, and even in high school, and it correlates with a variety of higher cognitive skills (Clements and Sarama, 2011). This issue captures the interest of research in the educational field.

Different neurological, cognitive, developmental, and educational approaches currently coexist, explaining why some children have an optimal mathematical development, whereas others have severe difficulties in the acquisition of the first mathematical skills (von Aster and Shalev, 2007; Castro-Cañizares et al., 2009; Geary, 2011; Ashkenazi et al., 2013). The variability in the results has too often been attributed to individual differences in intellectual capacity, although scores on the traditional IQ test have a limited influence on initial mathematical learning, based on the small percentage of variance shown by different studies (Veenman, 2006). Therefore, there is a growing body of multi-factorial approaches in which it is reported that mathematical performance occurs as a function of different factors and the interrelationships between them. This is the case of the Opportunity-Propensity model (Byrnes and Miller, 2007). According to this model, mathematical learning is the result of antecedent factors (related to the emergence of learning opportunities and propensities), opportunity factors (related with content exposure and teaching quality), and propensity factors (related to the ability or willingness to learn a content). Different studies on large secondary data-sets have revealed the value of this model in kindergarten (Byrnes and Wasik, 2009; Wang et al., 2013), the beginning of primary school (Byrnes and Wasik, 2009) and in secondary school (Byrnes and Miller, 2007, 2016). Byrnes and Wasik (2009) report that being exposed to learning opportunities is a necessary but not sufficient condition for mathematical performance. Children must have motivational disposition and prior cognitive skills (general and specific) to benefit from these opportunities, being these aspects especially important for the mathematical development. Thus, the complexity and the componential character of mathematical learning requires the consideration of different cognitive and motivational processes in any *ad hoc* explanatory model. A high priority in education is to clarify the cognitive and motivational processes involved and, above all, explain the interrelations between them.

### Early numeracy skills

The construction of number comprehension is one pillar of the higher order principles of mathematical learning that predict later mathematical outcomes beyond IQ variables (Baroody et al., 2006; Locuniak and Jordan, 2008). Numeracy, the core of formal mathematical thinking, requires a broad range of abilities that influence mathematical cognition development (Jordan et al., 2010; Stock et al., 2010). Within these “early numeracy skills,” three abilities can be stated as early and important components (Desoete and Grégoire, 2006): logical operations, counting, and magnitude comparsion abilities.

Among the logical operations, the seriation competence in particular has shown its effectiveness in predicting mathematical performance and detecting mathematical difficulties (Grégoire, 2005; Stock et al., 2007; Sarama and Clements, 2009; Aunio and Niemivirta, 2010; Desoete, 2014; Aunio et al., 2015; Tobia et al., 2016). Another precursor skill of formal mathematical knowledge is the capacity to count, which requires the assimilation of various logical principles: on the one hand, the understanding of ordinal numbers, and on the other hand, the understanding of the counting procedure. There is evidence of the fundamental role played by the early conceptual comprehension of counting principles in predicting mathematical difficulties (Jordan et al., 2010). Likewise, different longitudinal studies have demonstrated the influence of counting procedural knowledge in mathematical performance (Aunola et al., 2004; Jordan et al., 2007, 2009). In summary, counting consists of a cluster of concepts and skills that are mathematically central and coherent, consistent with children's thinking, and generative of future learning (Clements and Sarama, 2014).

Magnitude comparison ability is another skill different from “counting” and linked to early mathematical competence development and number understanding, which can be defined as the ability to determine the magnitude of a set without counting. It is a specific mechanism that we share with other species to represent numerical magnitudes, and it allows us to estimate the number of elements and the comparisons (Brannon, 2005; Butterworth, 2005; Wilson and Dehaene, 2007). Although there is currently no consensus on whether this ability is innate or not (Leibovich et al., 2017), a growing body of research has shown the relations between this early capacity, evaluated using comparison tasks, and math skills, both in early childhood education and in subsequent stages (De Smedt et al., 2009; Holloway and Ansari, 2009; Desoete et al., 2012; Libertus et al., 2013; Sasanguie et al., 2013).

### Executive functions

Executive functions are higher cognitive components that coordinate, regulate, and control cognitive processes during task performance (Miyake et al., 2000). The dynamic and complex nature of mathematical competence largely justifies the positive relations between EF, early numeracy skills, and later mathematical performance (Röthlisberger et al., 2013; Bull and Lee, 2014; Blair et al., 2016).

Different studies that have used factor analysis (exploratory and/or confirmatory) report that the structure of executive functioning has a marked developmental character. Thus, this construct tends to differentiate gradually over time (see Bull and Lee, 2014). In this sense, a recent review by Diamond (2013) suggests that inhibition and working memory (WM) are constituted as core components of executive functioning in the early stages of development, being the basis on which the other of executive functions lie.

Inhibition has shown a significant influence on mathematical performance during the early stages of development (Blair and Razza, 2007; Bull et al., 2008; Lan et al., 2011; Aragón et al., 2015). However, other investigations have not found these positive relations (van der Sluis et al., 2004; Censabella and Noël, 2008). A recent meta-analysis by Peng et al. (2015) included 110 studies that contributed to supporting the relation between working memory (WM) and mathematics. The data showed a correlation average of *r* = 0.35, which was similar in the different WM domains analyzed; however, differences appeared depending on the mathematical domain. WM had a stronger association with calculation and problem solving, but not with geometry. Differences emerged depending on the children's problems, with higher associations in children with mathematical difficulties and other unrelated disorders than in children with typical development or mathematical difficulties alone.

The goal of several studies was to have a deeper understanding of the relations between WM, early numeracy skills, and later mathematical performance. Thus, Toll et al. (2011) used a longitudinal design and found that WM predicted mathematical learning difficulties with a higher predictive value than early numeracy skills. Along the same lines, Östergren and Träff (2013) carried out a longitudinal study that analyzed the impact of early numeracy skills (naming Arabic digits; counting forward and backward; number line estimation task) and verbal WM (evaluated at the beginning of the last kidergarten course) on arithmetical competence (evaluated at the end of the course and a year later). The most significant conclusion was that both early numeracy skills and verbal WM affected arithmetical competence in the two assessments. However, verbal WM only had an indirect effect on competence in first grade, mediated by early numeracy skills and arithmetical competence in kidergarten. Passolunghi and Lanfranchi (2012), using path analysis models, also noticed the same indirect effect of WM in kidergarten on mathematical performance in first grade.

### Motivation

The executive functioning system, especially inhibition, can help children to regulate emotions and respond properly to failure and frustration. However, EF is not sufficient to persevere during a lifetime of mathematical learning. Mathematics learning and its difficulties also require great attention to motivational factors. These factors promote the effect of emotion regulation, cognitive strategies, and metacognitive strategies (Pintrich, 2003; Op't Eynde et al., 2006; Sarabia and Iriarte, 2011; Blair and Raver, 2015).

Motivation toward learning drives and maintains interest in challenges (Pintrich and Schunk, 2002; Wigfield and Eccles, 2002). Its involvement in mathematical performance starts from the first stage of education, and it affects the mathematical results in progressive years (Ladd et al., 2000; Mokrova et al., 2013; Reimann et al., 2013; Daniels, 2014; Presentación et al., 2015). Long-term growth in mathematical achievement is explained by motivational factors and by different strategies, but not by intelligence, even after controlling socio-demographic variables (Murayama et al., 2013).

In terms of learning behaviors, a major finding from the earliest models of motivation and behavior achievements is that when people expect to do well, they tend to try hard, persist, and perform better (Pintrich and Schunk, 2002). In fact, perceived competence, persistence to errors, or task perseverance in early childhood education have a significant impact on the prediction of later mathematical performance (Fantuzzo et al., 2004; Fitzpatrick and Pagani, 2013; Mercader et al., 2017). The capacity to anticipate success on the basis of perceived competence and persistence produces differences in the trajectories of children with and without mathematical performance problems. Perceived competence and persistence from kindergarten to the second year of Primary Education have been a protection factor against possible learning difficulties (McDermott et al., 2006, 2011, 2014; Mercader et al., 2017). These learning behaviors were shown to be protection factors in later stages of education and in multiple cultures (Chiu and Xihua, 2008).

Attributional style is another important motivational construct. It describes the subject's perception of the cause and effect of an event occurring to them and others (Weiner, 1986). People can attribute events to internal or external, stable or unstable, and specific or global causes, having implications for future behavior (Pintrich and Schunk, 2002). Attributing successful situations to internal and stable causes determines better school performance. On the other hand, attributing failure to internal, stable, and/or non-controllable causes would determine negative effects on future expectations of success and on final performance (Lozano et al., 2000; Manassero Mas and Vázquez, 2000; Pintrich and Schunk, 2002; González, 2005). Some attributional style dimensions are already present in early childhood education, as suggested by Presentación et al. (2015), who analyzed the predictive power of attributional style on initial mathematical skills. An interesting finding was that internality and stability of positive events predicted the ability to solve mathematical problems. A longitudinal study with the same sample indicated that internality of positive events made a significant contribution to explaining mathematical performance 2 years later (Mercader et al., 2017).

### The current study

After reviewing the literature, it can be concluded that research on mathematical learning has generally focused on partial aspects rather than on the comprehensive integration of executive procedures, motivation, and early mathematical skills. Advancing our knowledge involves understanding how different factors interact to generate different patterns of mathematical achievement. There is a great need for more longitudinal studies to analyze the complex and dynamic relations over time between motivation, executive functioning, early numeracy, and later mathematical outcomes.

Structural equation models would be a plausible pathway for this objective, given that, from this approach, each factor is an abstract concept that encompasses different observable variables measured individually and each of these factors has less error variance than each observable variable separately (Kline, 2016).

Based on the theoretical background and from a multifactorial and longitudinal approach, the model proposed in this study will include the main indicators of motivational constructs, executive functioning, and early numeracy skills assessed during early child education. The main goal is to examine the power of the model to predict mathematical performance in the second year of Primary Education. Basically, the model can be summarized in the following hypotheses:

1. The latent variable Executive Functioning is influenced by the latent variable Motivation.
2. The latent variable Early Numeracy Skills depends on the latent variables Motivation and Executive Functioning.
3. The latent variable Mathematical Performance (assessed in T2) is a function of the latent variables Motivation, Executive Functioning and Early Numeracy Skills.
4. The latent variables Executive Functioning and Early Numeracy Skills have a mediator effect between the latent Motivation variable and the latent variable Mathematical Performance.

**Figure 1:**
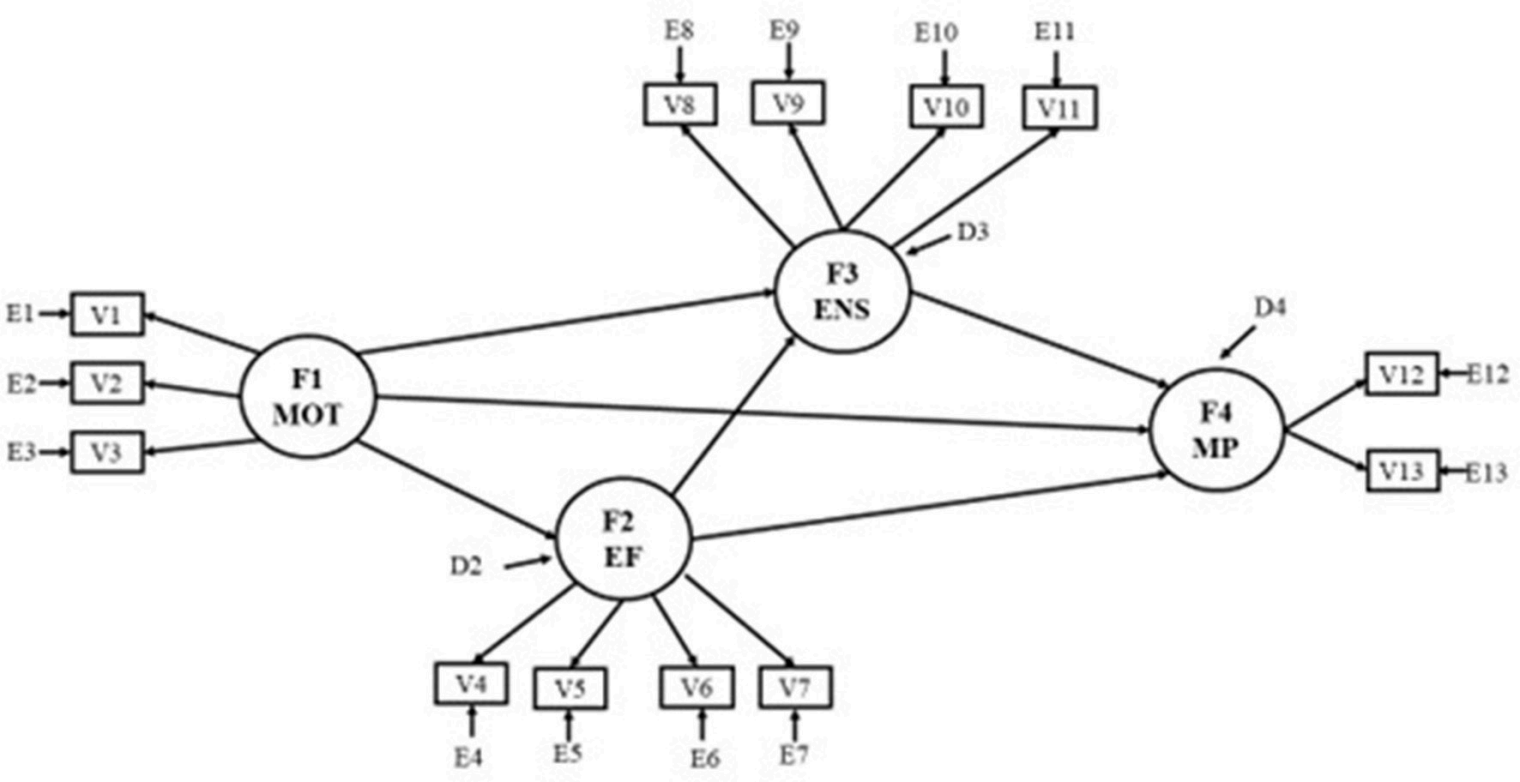
Hypothesized model. F1 MOT, Motivation; F2 EF, Executive Functioning; F3 ENS, Early Numeracy Skills; F4 MP, Mathematical Performance; V1, Competence-motivation; V2, Attention-persistence; V3, Positive internality; V4, Inhibition-Sun/Moon Stroop; V5, Inhibition-Tapping Task; V6, Working memory-Digit Span Backward; V7, Working memory-Counting Task; V8, Procedural counting; V9, Conceptual counting; V10, Logical operations; V11, Symbolic comparison; V12, Informal skills; V13, Formal skills.

Figure 1 shows the graphic representation of the structural equations model corresponding to the first three hypotheses presented above. The Bentler (2014) notation system was followed with regard to the variables, the prediction errors of the variables, the latent variables and disturbances, and the representation of the effects.

## Materials and methods

### Participants

First, in a wide range of schools, six students from each school were selected randomly through the simple random sampling technique (see Table 1). The criteria to participate in the study were as follows: IQ equivalent scores above 70; absence of problems such as autism spectrum disorder (ASD); sensory deficiencies; neurobiological abnormalities; serious psychological problems; chronic absenteeism; and/or lack of education as typically provided in the individual's community context.

**Table 1:** Sample description.

	**T1**	**T2**
**Sample size** [*n* (%)]	209 (100)	180 (86.6)
**GENDER**		
Male [*n* (%)]	109 (52.2)	92 (51.1)
Female [*n* (%)]	100 (47.8)	88 (48.9)
**AGE MONTHS** [mean (*SD*)]	70.17 (3.51)	94.16 (3.78)
**CENTER TYPE**		
Public Schools [*n* (%)]	133 (63.6)	117 (65)
Semi-private Schools [*n* (%)]	76 (36.4)	63 (35)
**EQUIVALENT IQ** [mean (*SD*)]	98.63 (12.23)	
**NATIONALITY**		
Spanish [*n* (%)]	184 (88)	158 (87.7)
Foreigner [*n* (%)]	24 (11.5)	22 (12.2)
**INTERVENTIONS WITH SPECIALISTS**		
Educational support [*n* (%)]	–	16 (7.7)
Compensatory education [*n* (%)]	–	4 (1.9)
Therapeutic pedagogy [*n* (%)]	–	7 (3.3)
Hearing and language [*n* (%)]	–	8 (3.8)
Combined treatment [*n* (%)]	–	5 (2.4)

In the initial sample, in the first data collection (T1) there were 209 kidergarten children in the last year (52.2% male; 47.8% girls), ranging from 5 to 6 years old (mean = 70.17 months; *SD* = 3.51 months). The children had an equivalent IQ average of 98.63 (*SD* = 12.23), obtained through the vocabulary and block design subtests (WPPSI, Wechsler, 1996), following the guidelines of Spreen and Strauss (1991). Among the children participating, 88% were Spanish, and the rest were from other countries. All subjects spoke and understood Spanish, regardless of their nationality; 63.6% of the children attended public schools, and the other 36.4 % were from semi-private schools.

Two years later (T2), 180 subjects (86.6% of the initial sample) were re-evaluated, with 13.4% experimental attrition. The participants in T2 ranged between 7 and 8 years old (mean = 94.16 months; *SD* = 3.78 months). Regarding gender, 51.1% of the subjects in the final sample were male, and 48.9% were female. In addition, 65% of the participants attended public schools, compared to 35% who belonged to semi-private schools. Moreover, 19.1% of participants attended interventions with specialists in their respective schools at T2: Educational support (7.7%), compensatory education (1.9%), therapeutic pedagogy (3.3%), hearing and language (3.8%), and combined treatment (2.4%).

### Instruments

#### Time 1: early childhood education−5 years

##### Executive functioning

The Sun-Moon Stroop Task (Archibald and Kerns, 1999) was used to evaluate inhibition through visual stimuli. This test consists of two conditions, congruent and incongruent. In the congruent condition, the subjects are shown a page with 30 pictures of the sun and moon placed randomly in rows and columns. Subjects have to respond “sun” to the images with the sun, and “moon” to the images with the moon, as quickly as possible (for 45 s). In the incongruent condition, subjects are asked to respond “sun” when the evaluator points to the moon, and “moon” when he/she points to the sun. This task has a high level of reliability, with test-retest scores of.91 for the incongruent condition (Archibald and Kerns, 1999). For the present study, Cronbach's alpha (α) was 0.94. To evaluate inhibition with auditory stimuli, Luria's Tapping Task was used (Luria, 1966). This task also consists of two conditions with 12 trials each. In the first, the subject has to repeat the same number of taps as the evaluator makes on the table (1 or 2), and then the subject has to do the opposite. Inhibition showed a task reliability of 0.87 (Diamond and Taylor, 1996). The α for the present study was 0.72. The interference measure is composed of the incongruent conditions on both tasks.

To evaluate verbal WM, two tasks were used, the Digit Span Task and the Working Memory-Counting Task. The Digit Span Task (Pickering et al., 1999) presents a series of from 2 to 9 digits (4 trials each). The task consists of repeating digits in the same sequence that the evaluator presents orally, but in an inverted order. The Working Memory-Counting Task (Case et al., 1982) consists of 3 levels (2–4 cards per level) with 4 trials in each. Each card contains blue and yellow dots arranged randomly. The subject has to state the number of blue dots on each card and remember them in the correct order once the series has been completed. Test-retest reliability was 0.64 (Alloway et al., 2006) and 0.62 (Gathercole et al., 2004), respectively. The reliability indicators for the present study were Digit Span Task, α = 0.70; Working Memory-Counting Task, α = 0.79. For both tasks, the sum of the correct trials is used for analysis.

##### Early numeracy skills

Kindergarten subtests from TEDI-MATH (Grégoire et al., 2005) were applied to assess the following math skills: Procedural counting (counting as high as possible, with a lower and upper limit, backwards, and by steps); conceptual counting (counting linear sets, random sets, isolation counted objects, and knowledge of cardinal numbers); logical operations (seriation, classification, conservation, and inclusion); and symbolic comparison (Arabic numeral comparison). The test has an internal consistency level ranging from 0.84 to 0.96, depending on each subtest and validity indexes (Grégoire et al., 2005). The reliability indicators for the present study were procedural counting, α = 0.83; conceptual counting, α = 0.67; logical operations, α = 0.67; symbolic comparison, α = 0.80. The correct answers in each domain were used.

##### Motivation

Teachers completed the Preschool Learning Behaviors Scale (PLBS; McDermott et al., 2000), designed to identify motivational behaviors toward preschool-aged students on a Likert-type scale (0 = “very often,” 1 = “sometimes,” 2 = “almost never”). The PLBS comprises 29 items grouped in three subscales: competence-motivation, attention-persistence, and learning attitude. Competence-motivation and attention-persistence were used in the present study. Competence-motivation is related to success anticipation (e.g., “He/she takes refuge in a powerlessness attitude”) and attention-persistence is focused on the ability to persist on a task until it is completed (e.g., “He/she is involved in the tasks to the extent expected for his/her age”). The reliability indicators for the present study are: competence-motivation: α = 0.89, Composite reliability (CR) = 0.90, Average Variance Extracted (AVE) = 0.87, McDonald's Omega (Ω) = 0.84; attention-persistence: α = 0.85, CR = 0.86, AVE = 0.86, Ω = 0.80. Competence-motivation and attention-persistence raw scores were used for the analysis.

Likewise, the Children's Attributional Style Interview (CASI; Conley et al., 2001) was administered individually. This interview is applicable to children aged 5 or more. On this task, performance-related events were used to illustrate 16 stories to the subjects (e.g., cognitive tasks, school situations, sports, etc.). The child had to rate them in terms of internality (1 = “it depends on me” vs. 0 = “it depends on others”), globality (1 = “it happens everywhere” vs. 0 = “it only happens in a specific situation”), and stability (1 = “it happens a lot” vs. 0 = “it only happens this time”). Half of the stories are positive, and the rest are negative. The alternatives were counter-balanced to minimize interference in the responses. The reliability indicators for the test in the present sample are: α = 0.56, CR = 0.63, AVE = 0.59, Ω = 0.77. Only internality responses to positive events were used for referencing.

#### Time 2: 2nd year of primary education

##### Mathematical performance

To evaluate mathematical performance, the Test of Early Mathematics Ability (TEMA-3; Ginsburg and Baroody, 2003) was applied. It is a standardized test designed for use with children from 3 years old to 8 years and 11 months. The goal of the test is to identify specific strengths and weaknesses in mathematical competence. It consists of 72 items evaluating different areas of mathematical competence. The test covers informal abilities evaluated by 41 items (those that do not require the use of written mathematical symbols), and formal skills grouped in 31 items (activities that involve the use of mathematical symbols). The skills are grouped in 8 dimensions: Four subscales related to the informal skills of numeration, comparison, calculation, and concepts; four subscales of formal skills on conventionalisms, numerical facts, calculation, and concepts. The Spanish version of the test has levels of reliability of 0.94 and 0.91 for samples of 7 and 8 years, respectively (Ginsburg and Baroody, 2003). The α for the present study were 0.80 and 0.85, respectively. The sum of the informal and formal skills subscale scores (separately) was used in this study.

### Procedure

The present study was carried out in accordance with the Helsinki declaration (1964). The study was approved by the Ethics Committee of Jaume I University and the official and written permissions of the Board of Education and School Management was obtained. Written and informed consent from the parents/legal guardians and teachers of all participants were obtained. Participant's oral consent was also obtained.

The assessment was carried out by trained professionals. The administration was individual, respecting individual rhythms. The physical spaces fulfilled the conditions of lighting, ventilation, and soundproofing suitable for the evaluation.

In T1, neuropsychological tasks of inhibition and WM, TEDI-MATH (Grégoire, 2005), and CASI (Conley et al., 2001) were administered in two individual sessions lasting ~30 min each. The evaluation was carried out at the school, without interfering with the normal curriculum, during the second semester of the course. In addition, teachers completed the PLBS scale (McDermott et al., 2000).

Two years later, at T2, TEMA-3 (Ginsburg and Baroody, 2003) was administrated to the same subjects, following the same procedure described above.

### Data analysis

The listwise deletion procedure was used for the treatment of missing values. Structural equation modeling (SEM) was used to verify the structural organization of the theoretical model shown in Figure 1, using the EQS v6.2 program (Bentler, 2014). The Mardia multivariate kurtosis test (1970) yielded a high value (23.16). Keeping this in mind, a structural equations analysis was performed using the robust estimation method of maximum likelihood (Satorra and Bentler, 1994; Bentler, 2014). Fit indexes proposed by Bentler (1990) were used (see Bentler, 1990 to see the technical and statistical differences between fit indixes employed).

## Results

### Model contrast

As a criterion for using the possible adjustment acceptance of a structural equation model, the orientations of Kline (2016) and Little (2013) were used. Thus, the Satorra-Bentler Robust Chi-square ($\chi_{SB}^{2}$) should have a *p* > 0.05. But, because it depends on the sample size, the Relative Chi-square ($\left(\chi_{SB}^{2}/df\right)$ is used. This means the division of the Chi-square between its freedom degrees (< 2:1). The Bentler-Bonnet Normed Fit Index (NFI), the Bentler-Bonnet Nonnormed Fit Index (NNFI), the Comparative Fit Index (CFI), the Bollen's Fit Index (BFI), and the McDonal's Fit Index (MDFI) are also used. They should be 0.85 and 0.90 to be considered poor, between 0.90 and 0.95 to be acceptable, between 0.95 and 0.99 to be very good, and >0.99 to be outstanding. The value of the Root Mean Square Error of Approximation (RMSEA), should be between 0.10 and 0.08 to be considered poor fit, between 0.08 and 0.05 to be an acceptable fit, between 0.05 and 0.02 to be a good fit, and it is considered great fit if <0.02. The reliability indicators for each latent variable were tested [Motivation (F1), α = 0.93; Executive functioning (F2), α = 0.94; Early numeracy skills (F3), α = 0.86; Mathematic Performance (F4), α = 0.93].

**Figure 2:**
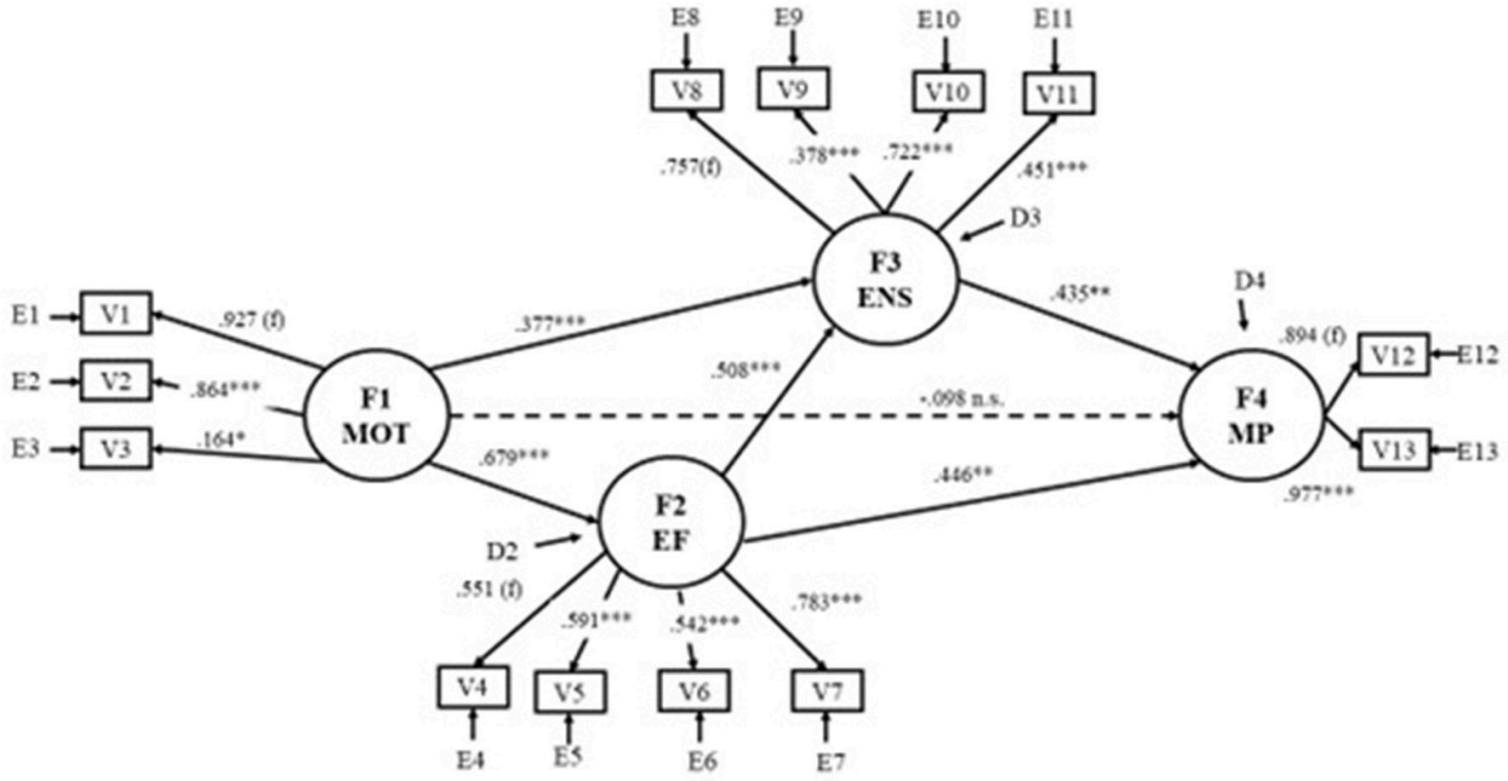
Results of the effects (in standardized scores) of the initial model proposed in Figure 1. F1 MOT, Motivation; F2 EF, Executive Functioning; F3 ENS, Early Numeracy Skills; F4 MP, Mathematical Performance; V1, Competence-motivation; V2, Attention-persistence; V3, Positive internality; V4, Inhibition-Sun/Moon Stroop; V5, Inhibition-Tapping Task; V6, Working memory-Digit Span Backward; V7, Working memory-Counting Task; V8, Procedural counting; V9, Conceptual counting; V10, Logical operations; V11, Symbolic comparison; V12, Informal skills; V13, Formal skills. *f*, fixed effect; ^*^p < 0.05; ^**^p < 0.01; ^***^p < 0.001; n.s., non-significant.

The results of the operationalized theoretical model represented in Figure 1 are shown in Figure 2. Using the fit indices, the final model was found to fit the data: $\chi_{SB}^{2}$ = 95.86, 59 df, *p* = 0.002; $\chi_{SB}^{2}/df)$ = 1.62; NFI = 0.902; NNFI = 0.947; CFI = 0.960; BFI = 0.961; MDFI = 0.905; RMSEA = 0.058.

The effects of the relations between the latent variables are significant, with the exception of the effect of the latent variable Motivation (T1) on Mathematic Performance (T2). Therefore, the model in Figure 2 represents the relations among the variables in the most parsimonious way.

The latent variable Mathematic Performance (F4) receives significant effects only from the latent variables Executive Functioning (F2) and Early Numeracy Skills (F3), but not from Motivation (F1). In this way, hypothesis 1–3 are confirmed, except the effect of the latent variable Motivation on the latent variable Mathematic Performance. The explained variance of the model dependent variable (F4, Mathematic Performance) was *R*^2^ = 0.573.

### Indirect and mediation effects analysis

**Figure 3:**
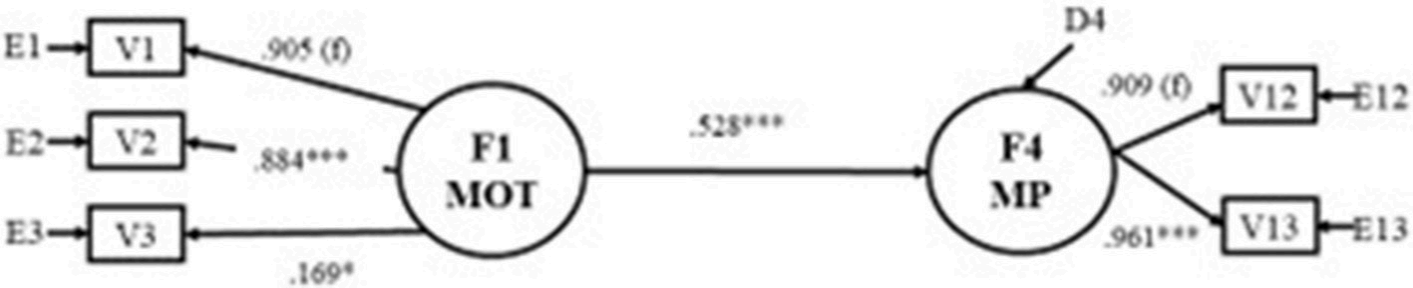
Total effect (in standardized scores) of F1 on F4. F1MOT, Motivation; F4MP, Mathematical Performance; V1, Competence-motivation; V2, Attention-persistence; V3, Positive internality; V12, Informal skills; V13, Formal skills. *f*, fixed effect; ^*^p < 0.05; ^***^p < 0.001.

Due to the not significant influence of latent Motivation (F1) on Mathematical Performance (F4) in the model (Figure 2), the mediation effect of the latent variables Executive Functioning (F2) and Early Numeracy Skills (F3) on the relation of Motivation (F1) and Mathematic Performance (F4) were tested. Following the guidelines of Baron and Kenny (1986), the analysis of the total effect regarding the relation between Motivation (F1) and Mathematic Performance (F4) was conducted (Figure 3). The effect of Motivation (F1) on Mathematic Performance (F4) is significant, and the model was found to fit the data: $\chi_{SB}^{2}$ = 5.26, df, 4 *p* = 0.261; $\chi_{SB}^{2}$/df = 1.31; NFI = 0.989; NNFI = 0.993; CFI = 0.997; BFI = 0.997; MDFI = 0.996; RMSEA = 0.042. The latent variable Mathematic Performance (F4) receives a significant effect of the latent variable Motivation (F1).

On the other hand, Sobel Test (1982) was conducted separately to check the significance of the indirect effects included in the model. The indirect effects of the latent variable Motivation (F1) on the latent variable Mathematic Performance (F4) were significant both in the latent variable Executive Functioning (F2) (Sobel Test = 3.68; SE = 0.09; *p* < 0.001) and in the latent variable Early Numeracy Skills (F3) (Sobel Test = 4.52; SE = 0.07; *p* < 0.001). The indirect effect between the latent variables Executive Functioning (F2) and Mathematic Performance (F4) through the latent variable Early Numeracy Skills (F3) was also statistically significant (Sobel Test = 2.38; SE = 0.10; *p* = 0.02). The results set suggest the confirmation of the hypothesis 4, so that there is an indirect effect of motivation on later mathematical performance, mediated by executive functioning and the early numeracy skills. The apparent effect of motivation on later mathematical performance is due to the effect of motivation on executive functioning and on early numeracy skills and in turn these two latent variables on the mathematic performance.

## Discussion

### Summary of study purpose and goals

A longitudinal study was carried out to analyze the complex relations among motivation, executive functioning, early numeracy skills, and later mathematical outcomes. To achieve this goal, in the initial stages of learning, the main indicators of these constructs were measured. Specifically, the objective was to analyze the capacity of motivation, EF, and early numeracy skills assessed in Early Education to predict mathematical performance in the second year of Primary Education using structural equation modeling. This strategy would seem to be more fruitful than attempts to prove the importance of single constructs in isolation.

### Findings and interpretations

Results show that together motivation, EF, and early numeracy skills explain 57.3% of the variance in later mathematical performance. As expected, the analysis showed a close relation between early numeracy skills and later mathematical performance, agreeing with different studies that highlight a specific contribution of counting, procedural knowledge, and logical operations (Aunola et al., 2004; Grégoire, 2005; Jordan et al., 2007, 2009; Stock et al., 2007; Aunio and Niemivirta, 2010; Tobia et al., 2016).

The contrasted model also confirmed that the EF, evaluated with inhibition and WM tests in kidergarten, has a significant effect on both early numeracy skills and mathematical performance in Primary School. These results are consistent with other studies that have analyzed these relations independently (Blair and Razza, 2007; Bull et al., 2008; Lan et al., 2011; Aragón et al., 2015; Peng et al., 2015). Toll et al. (2011), in children with difficulties, also showed this double influence of EF. This seems to reinforce the hypothesis of a higher importance of the EF, compared to early numeracy skills, in explaining later mathematical performance.

However, this double effect of EF on mathematical skills at both moments does not appear in longitudinal studies analyzed with structural equations and path analysis (Passolunghi and Lanfranchi, 2012; Östergren and Träff, 2013). These investigations report only an indirect effect of WM on later mathematical performance, mediated by early numeracy skills. This disparity in the results could be related to the older age of the subjects and/or the absence of inhibition measures.

One aspect that deserves special interest, given the little research on the topic, is related to the explanatory power of motivational variables. In this model, it is clear that the EF are influenced by motivation, confirming the first hypothesis. The results of this study defend the presence of cognitive regulation based on motivational variables from early stages, similar to other past studies (Pintrich, 2003; Sarabia and Iriarte, 2011; Blair and Raver, 2015). The contrasted model also highlights that motivation (along with EF) has a significant effect on early numeracy skills (thus confirming the second proposed hypothesis). These results match other studies that have analyzed these relations in isolation (Ladd et al., 2000; Mokrova et al., 2013; Reimann et al., 2013; Daniels, 2014; Presentación et al., 2015).

The third hypothesis was not completely confirmed, but some relations have been found. A direct effect of initial motivation toward learning on later mathematical performance was not found. This result contrasts with longitudinal studies that have investigated the relations between the same variables (Miñano and Castejón, 2011; Cerda et al., 2015). Nevertheless, these studies have not valued the role of general early motivation toward learning, but rather specific motivation toward mathematics in higher education cycles. Motivation in this developmental stage would possibly result from numerous previous experiences of success and/or failure in mathematical activities, which would explain its close and direct relation with later mathematical performance.

The absence of a direct relation between motivation and later performance in the model is also inconsistent with research that has studied this relation in isolation, and it shows the importance of variables such as perceived competence or persistence in predicting later mathematical performance (Fantuzzo et al., 2004; Fitzpatrick and Pagani, 2013; Mercader et al., 2017). Examining this point more closely, the relation between initial motivation and later mathematical performance has been analyzed separately, showing a direct effect. However, when including EF and early numeracy skills in the model, the relation is indirect, thus confirming the fourth hypothesis. Specifically, initial motivation for learning and attributions in kindergarten, particularly perceived competence and persistence, are highly related to later mathematical competence in three ways. First, the model shows that motivation has a significant effect on later mathematical outcomes mediated by EF. Second, motivation affects later mathematical outcomes through early numeracy skills. Finally, it is clear that there is a relation between motivation and later mathematical competence through the EF and early numeracy skills.

### Implications for practice

Our results could have extensive educational implications, stressing that learning is not reduced to the mere acquisition of knowledge, but it must also promote the internalization of a positive attitude toward the task. Developing EF and mathematical competencies promotes engagement, but this is not the only way to engage students. Along with the development of EF and early numeracy skills, it is important for educators and parents to enhance children's motivation as a basic driver of learning, both at home and in school settings.

Some educators worry that structured math activities will negatively influence children's motivation. We do not know of research supporting this concern; however, there has been research that suggests the opposite. Motivation and engagement increase with intentional, structured, and appropriate math activities. Educators have to avoid narrow views of math and learning. Teachers hamper students' learning when they define success as fast, correct, and accurate responses following the teacher's example. Mathematics are experienced as compartmentalized pieces with no meaning as a tool for problem solving in daily life (see Turner and Meyer, 2009). Motivation promotes meaningful learning, unlike rote, and repetitive learning. In this regard, educators should promote and reinforce adaptive perceptions of competence and control, and promote academic values and aims. Thus, interesting mathematical tasks such as projects that emerge from student's interests based on mathematical lessons and discussions about children's ideas/strategies, activities focused on higher-order thinking, and avoiding anxiety by promoting cooperative work rather than competitive, are essential teaching practices in mathematical learning. Furthermore, motivation in mathematics cannot be understood without connections to students' lives and cultures. This is a key point in creating a sense of belonging and engaging in active participation to promote motivation toward mathematics. Finally, these critical points should be implemented from the earliest stages of education, given their implications in later mathematical performance.

### Strengths and limitations of this study

The current study has important strengths that include the large sample of kindergarten children, the use of a longitudinal design, and assessment of diverse qualitative factors involved in mathematical performance. It can be concluded that all the variables analyzed have shown a specific direct or indirect importance in the explanation of later mathematical competence. Nevertheless, this study has some limitations. Future longitudinal studies analyzing precursors of mathematical achievement should also consider other personal factors that have not been included in this research (see Byrnes and Miller, 2007). For example, EF as planning or monitoring, or other variables such as motivational achievement goal orientations should be included in future research. Neither have in our study indicators of linguistic precursors, such as phonological awareness or rapid naming, which have demonstrated their ability to predict performance in calculation tasks (Cirino, 2011). Variables related to the family that have not been included would provide a more complete model (e.g., parents' socio-economic status or types of child-rearing strategies). Finally, there is a lack of instructional factors, such as the type and amount of teaching to which the children were exposed, which could be related to the amount of explained variance in the model.

## Conclusion

Progress in the research on mathematical education involves understanding how different factors interact to lead to mathematical achievement. In this longitudinal study, executive functioning, motivation, and early mathematical skills are assessed to explain later mathematical performance. Structural equations analysis showed a direct effect of executive functioning and early numeracy skills on later mathematical performance. Initial motivation did not have a significant direct effect on mathematical performance. Motivation had an indirect effect through executive functioning, early numeracy skills, and their combination. Educators should promote motivation from early stages of education, given its importance in later success in learning.

## Author contributions

AM: Substantial contributions to the conception or design of the work; revising the work critically for important intellectual content. JM, MP, RS: Acquisition and interpretation of data for the work; drafting and revising the work. JR: Analysis and interpretation of the data.

### Conflict of interest statement

The authors declare that the research was conducted in the absence of any commercial or financial relationships that could be construed as a potential conflict of interest.

## References

[B1] Alloway T. P. Gathercole S. E. Pickering S. J. (2006). Verbal and visuospatial short-term and working memory in children: Are they separable? Child. Dev. 77, 1698–1716. 10.1111/j.1467-8624.2006.00968.x 17107455

[B2] Ancker J. S. Kaufman D. (2007). Rethinking health numeracy: a multidisciplinary literature review. J. Am. Med. Inf. Assoc. 14, 713–721. 10.1197/jamia.M2464 17712082 PMC2213486

[B3] Aragón E. L. Guzmán J. I. N. Villagrán M. A. Cerda G. (2015). Predictores cognitivos del conocimiento numérico temprano en alumnado de 5 a-os [Cognitive predictors of early numerical knowledge in 5-year-old pupils]. Rev. Psicod. 20, 83–97. 10.1387/RevPsicodidact.11088

[B4] Archibald S. J. Kerns K. A. (1999). Identification and description of new tests of executive functioning in children. Child. Neuropsychol. 5, 115–129. 10.1076/chin.5.2.115.3167

[B5] Ashkenazi S. Black J. M. Abrams D. A. Hoeft F. Menon V. (2013). Neurobiological underpinnings of math and reading learning disabilities. J. Learn. Dis. 46, 549–569. 10.1177/0022219413483174 23572008 PMC3795983

[B6] Aunio P. Heiskari P. Van Luit J. Vuorio J. M. (2015). The development of early numeracy skills in kindergarten in low-, average, and high performance groups. J. Early. Child. Res. 20, 1–14. 10.1177/1476718X14538722

[B7] Aunio P. Niemivirta M. (2010). Predicting children's mathematical performance in grade one by early numeracy. Learn. Individ. Diff. 20, 427–435. 10.1016/j.lindif.2010.06.003

[B8] Aunola K. Leskinen E. Lerkkanen M. K. Nurmi J. E. (2004). Developmental dynamics of math performance from preschool to grade 2. J. Educ. Psychol. 96, 699–713. 10.1037/0022-0663.96.4.699

[B9] Baron R. M. Kenny D. A. (1986). The moderator-mediator variable distinction in social psychological research: conceptual, strategic and statistical considerations. J. Pers. Soc. Psychol. 51, 1173–1182. 10.1037/0022-3514.51.6.1173 3806354

[B10] Baroody A. J. Lai M.-L. Mix K. S. (2006). The development of young children's number and operation sense and its implications for early childhood education, in Handbook of Research on the Education of Young Children, eds Spodek B. Saracho O. (Mahwah, NJ: Erlbaum),187–221.

[B11] Bentler P. M. (1990). Comparative fit indexes in structural models. Psychol. Bull. 107, 218–246. 10.1037/0033-2909.107.2.238 2320703

[B12] Bentler P. M. (2014). EQS Structural Equations Program Manual. Encino, CA: Multivariate Software.

[B13] Blair C. McKinnon R. D. Family Life Project Investigators (2016). Moderating effects of executive functions and the teacher–child relationship on the development of mathematics ability in kindergarten. Learn. Instruct. 41, 85–93. 10.1016/j.learninstruc.2015.10.001 28154471 PMC5283384

[B14] Blair C. Raver C. C. (2015). School readiness and self-regulation: a developmental psychobiological approach. Annu. Rev. Psychol. 66, 711–731. 10.1146/annurev-psych-010814-015221 25148852 PMC4682347

[B15] Blair C. Razza R. P. (2007). Relating effortful control, executive function, and false belief understanding to emerging math and literacy ability in kindergarten. Child. Dev. 78, 647–663. 10.1111/j.1467-8624.2007.01019.x 17381795

[B16] Brannon E. M. (2005). What animals know about numbers, in Handbook of Mathematical Cognition, ed Campbell J. I. (New York, NY: Psychology Press), 85–107.

[B17] Bull R. Espy K. A. Wiebe S. A. (2008). Short-term memory, working memory, and executive functioning in preschoolers: longitudinal predictors of mathematical achievement at age 7 years. Dev. Neuropsychol. 33, 205–228. 10.1080/87565640801982312 18473197 PMC2729141

[B18] Bull R. Lee K. (2014). Executive functioning and mathematics achievement. Child Dev. Perspect. 8, 36–41. 10.1111/cdep.12059

[B19] Butterworth B. (2005). The development of arithmetical abilities. J. Child Psychol. Psychiatry 46, 3–18. 10.1111/j.1469-7610.2004.00374.x 15660640

[B20] Byrnes J. P. Miller D. C. (2007). The relative importance of predictors of math and science achievement: an opportunity–propensity analysis. Contemp. Educ. Psychol. 32, 599–629. 10.1016/j.cedpsych.2006.09.002

[B21] Byrnes J. P. Miller D. C. (2016). The growth of mathematics and reading skills in segregated and diverse schools: an opportunity-propensity analysis of a national database. Contemp. Educ. Psychol. 46, 34–51. 10.1016/j.cedpsych.2016.04.002

[B22] Byrnes J. P. Wasik B. A. (2009). Factors predictive of mathematics achievement in kindergarten, first and third grades: an opportunity–propensity analysis. Contemp. Educ. Psychol. 34, 167–183. 10.1016/j.cedpsych.2009.01.002

[B23] Case R. Kurland D. M. Goldberg J. (1982). Operational efficiency of a short-term memory span. J. Exp. Psychol. 33, 386–404. 10.1016/0022-0965(82)90054-6

[B24] Castro-Cañizares D. Estévez-Pérez N. Reigosa-Crespo V. (2009). Teorías cognitivas contemporáneas sobre la discalculia del desarrollo [Contemporary cognitive theories about developmental dyscalculia]. Rev. Neurol. 49, 143–148. 19621309

[B25] Censabella S. Noël M. P. (2008). The inhibition capacities of children with mathematical disabilities. Child Neuropsychol. 14, 1–20. 10.1080/09297040601052318 18097799

[B26] Cerda G. Pérez C. Navarro J. I. Aguilar M. Casas J. A. Aragón E. (2015). Explanatory model of emotional-cognitive variables in school mathematics performance: a longitudinal study in primary school. Front. Psychol. 6:1363. 10.3389/fpsyg.2015.01363 26441739 PMC4561756

[B27] Chiu M. M. Xihua Z. (2008). Family and motivation effects on mathematics achievement: analyses of students in 41 countries. Learn. Inst. 18, 321–336. 10.1016/j.learninstruc.2007.06.003

[B28] Cirino P. T. (2011). The interrelationships of mathematical precursors in kindergarten. J. Exp. Child Psychol. 108, 713–733. 10.1016/j.jecp.2010.11.004 21194711 PMC3043138

[B29] Clements D. H. Sarama J. (2011). Early childhood mathematics intervention. Science 333, 968–970. 10.1126/science.1204537 21852488

[B30] Clements D. H. Sarama J. (2014). Learning and Teaching Early Maths. The Learning Trajectories Approach. New York, NY: Taylor & Francis.

[B31] Conley C. S. Haines B. A. Hilt L. M. Metalsky G. I. (2001). Children's Attributional Style Interview. Washington, DC: PsycTESTS. 10.1023/a:1010451604161 11695545

[B32] Daniels D. H. (2014). Children's affective orientations in preschool and their initial adjustment to kindergarten. Psychol. Schools 51, 256–272. 10.1002/pits.21748

[B33] De Smedt B. Verschaffel L. Ghesquière P. (2009). The predictive value of numerical magnitude comparison for individual differences in mathematics achievement. J. Exp. Child. Psychol. 103, 469–479. 10.1016/j.jecp.2009.01.010 19285682

[B34] Desoete A. (2014). Predictive indicators for mathematical learning disabilities/dyscalculia in kindergarten children, in The Routledge International Handbook of Dyscalculia and Mathematical Learning Difficulties, ed Chinn S. (Oxford: Routledge), 90–100.

[B35] Desoete A. Ceulemans A. De Weerdt F. Pieters S. (2012). Can we predict mathematical learning disabilities from symbolic and non-symbolic comparison tasks in kindergarten? Findings from a longitudinal study. Br. J. Educ. Psychol. 82, 64–81. 10.1348/2044-8279.002002 21199482

[B36] Desoete A. Grégoire J. (2006). Numerical competence in young children and in children with mathematics learning disabilities. Learn. Indiv. Diff. 16, 351–367. 10.1016/j.lindif.2006.12.006

[B37] Diamond A. (2013). Executive functions. Annu. Rev. Psychol. 64, 135. 10.1146/annurev-psych-113011-143750 23020641 PMC4084861

[B38] Diamond A. Taylor C. (1996). Development of an aspect of executive control: development of the abilities to remember what I said and to “Do as I say, not as I do”. Dev. Psychobiol. 29, 315–334. 10.1002/(SICI)1098-2302(199605)29:4<315::AID-DEV2>3.0.CO;2-T 8732806

[B39] Fantuzzo J. Perry M. A. McDermott P. (2004). Preschool approaches to learning and their relationship to other relevant classroom competencies for low-income children. Sch. Psychol. Q. 19, 212–230. 10.1521/scpq.19.3.212.40276

[B40] Fitzpatrick C. Pagani L. S. (2013). Task-oriented kindergarten behavior pays off in later childhood. J. Dev. Behav. Pediatr. 34, 94–101. 10.1097/DBP.0b013e31827a3779 23369956

[B41] Gathercole S. E. Pickering S. J. Ambridge B. Wearing H. (2004). The structure of working memory from 4 to 15 years of age. Dev. Psychol. 40, 177–190. 10.1037/0012-1649.40.2.177 14979759

[B42] Geary D. C. (2011). Consequences, characteristics, and causes of mathematical learning disabilities and persistent low achievement in mathematics. J. Dev. Behav. Pediatr. 32, 250–263. 10.1097/DBP.0b013e318209edef 21285895 PMC3131082

[B43] Ginsburg H. Baroody A. (2003). TEMA-3; Test de Competencia Matemática Básica, 3ª Edición [Tema-3; Test of Early Mathematics Ability, 3rd Edition]. Madrid: TEA Ediciones.

[B44] González A. (2005). Motivación Académica: Teoría, Aplicación y Evaluación [Academic Motivation: Theory, Application and Assessment]. Madrid: Pirámide.

[B45] Grégoire J. (2005). Logical development and arithmetic competency: is the Piagetan model always current?, in Enseignement et Appretissage des Mathématiques, eds Crahay M. Verschaffel L. Grégoire J. (Bruxelles: De Boeck), 57–77.

[B46] Grégoire J. Nöel M. Van Nieuwenhoven C. (2005). TEDI-MATH; Test para el Diagnostico de las Competencias Básicas en Matemáticas [TEDI-MATH; Test for the Diagnosis of Basic Competences in Mathematics]. Madrid: TEA Ediciones.

[B47] Holloway I. D. Ansari D. (2009). Mapping numerical magnitudes onto symbols: the numerical distance effect and individual differences in children's mathematics achievement. J. Exp. Child Psychol. 103, 17–29. 10.1016/j.jecp.2008.04.001 18513738

[B48] Jordan N. C. Glutting J. Ramineni C. (2010). The importance of number sense to mathematics achievement in first and third grades. Learn. Individ. Diff. 20, 82–88. 10.1016/j.lindif.2009.07.004 20401327 PMC2855153

[B49] Jordan N. C. Kaplan D. Locuniak M. N. Ramineni C. (2007). Predicting first-grade math achievement from developmental number sense trajectories. Learn. Disabil. Res. Pract. 22, 36–46. 10.1111/j.1540-5826.2007.00229.x

[B50] Jordan N. C. Kaplan D. Ramineni C. Locuniak M. N. (2009). Early math matters: kindergarten number competence and later mathematics outcomes. Dev. Psychol. 45, 850–867. 10.1037/a0014939 19413436 PMC2782699

[B51] Kline R. B. (2016). Principles and Practice of Structural Equation Modeling, 4th Edn. New York, NY: Guilford.

[B52] Ladd G. W. Bush E. S. Seid M. (2000). Children's initial sentiments about kindergarten: is school liking an antecedent of early classroom participation and achievement? Merrill Palmer Q. 46, 255–279.

[B53] Lan X. Legare C. H. Ponitz C. C. Li S. Morrison F. J. (2011). Investigating the links between the subcomponents of executive function and academic achievement: a cross-cultural analysis of Chinese and American preschoolers. J. Exp. Child Psychol. 108, 677–692. 10.1016/j.jecp.2010.11.001 21238977

[B54] Leibovich T. Katzin N. Harel M. Henik A. (2017). From “sense of number” to “sense of magnitude”: the role of continuous magnitudes in numerical cognition. Behav. Brain Sci. 40:e164 10.1017/S0140525X16000960 27530053

[B55] Libertus M. E. Feigenson L. Halberda J. (2013). Is approximate number precision a stable predictor of math ability? Learn. Individ. Diff. 25, 126–133. 10.1016/j.lindif.2013.02.001 23814453 PMC3692560

[B56] Little (2013). Longitudinal Structural Equation Modeling. New York, NY: Guilford.

[B57] Locuniak M. N. Jordan N. C. (2008). Using kindergarten number sense to predict calculation fluency in second grade. J. Learn. Disabil. 41, 451–459. 10.1177/0022219408321126 18768776 PMC3935894

[B58] Lozano A. B. Pesutti C. R. Blanco J. C. B. Canosa S. S. (2000). Factores de atribución causal, enfoques de aprendizaje y rendimiento académico en el alumnado de educación secundaria de Galicia: datos para un análisis correlacional [Causal attribution factors, learning approaches and academic performance in secondary education students of Galicia: data for a correlation analysis]. Rev. Estud. Invest. Psicol. Educ. 6, 792.

[B59] Luria A. R. (1966). Higher Cortical Functions in Man. New York, NY: Basic Books.

[B60] Manassero Mas M. A. Vázquez A. (2000). Análisis empírico de dos escalas de motivación escolar [Empirical analysis of two scales of school motivation]. REME. 3, 5–6.

[B61] McCloskey M. (2007). Quantitative literacy and developmental dyscalculias, in Why is Math So Hard For Some Children? The Nature and Origins of Children's Mathematical Learning Difficulties and Disabilities, eds Berch D. B. Mazzocco M. M. (Baltimore, MD: Brookes), 415–429.

[B62] McDermott P. A. Fantuzzo J. W. Warley H. P. Waterman C. Angelo L. E. Gadsden V. L. (2011). Multidimensionality of teachers' graded responses for preschoolers' stylistic learning behavior: the Learning-To-Learn Scales. Educ. Psychol. Meas. 71, 148–169. 10.1177/0013164410387351

[B63] McDermott P. A. Goldberg M. M. Watkins M. W. Stanley J. L. Glutting J. J. (2006). A nationwide epidemiologic modeling study of LD risk, protection, and unintended impact. J. Learn. Disabil. 39, 230–251. 10.1177/00222194060390030401 16724795

[B64] McDermott P. A. Green L. F. Francis J. M. Stott D. H. (2000). PLBS; Preschool Learning Behaviors Scale. Philadelphia, PA: Edumetric & Clinical Science.

[B65] McDermott P. A. Rikoon S. H. Fantuzzo J. W. (2014). Tracing children's approaches to learning through Head Start, kindergarten, and first grade: different pathways to different outcomes. J. Educ. Psychol. 106, 200–213. 10.1037/a0033547

[B66] Mercader J. Presentación M. J. Molinero V. Siegenthaler R. Miranda A. (2017). Motivación y rendimiento académico en matemáticas: un estudio longitudinal en las primeras etapas educativas [Motivation and academic achievement in mathematics: a longitudinal study in the early stages of education]. Rev. Psic. 22, 157–163. 10.1016/j.psicod.2017.05.007

[B67] Miñano P. Castejón J. L. (2011). Variables cognitivas y motivacionales en el rendimiento académico en Lengua y Matemáticas: un modelo estructural [Cognitive and motivational variables in academic performance in language and mathematics: a structural model]. Rev. Psic. 16, 203–230.

[B68] Miyake A. Friedman N. P. Emerson M. J. Witzki A. H. Howerter A. Wager T. D. (2000). The unity and diversity of executive functions and their contributions to complex “frontal lobe” tasks: a latent variable analysis. Cogn. Psychol. 41, 49–100. 10.1006/cogp.1999.0734 10945922

[B69] Mokrova I. L. O'Brien M. Calkins S. D. Leerkes E. M. Marcovitch S. (2013). The role of persistence at preschool age in academic skills at kindergarten. Eur. J. Psychol. Educ. 28, 1495–1503. 10.1007/s10212-013-0177-2

[B70] Murayama K. Pekrun R. Lichtenfeld S. vom Hofe R. (2013). Predicting long-term growth in students' mathematics achievement. The unique contribution of motivation and cognitive strategies. Child Dev. 84, 1475–1490. 10.1111/cdev.12036 23278807

[B71] Op't Eynde P. De Corte E. Verschaffel L. (2006). “Accepting emotional complexity”: a socio-constructivist perspective on the role of emotions in the mathematics classroom. Educ. Stud. Math. 63, 193–207. 10.1007/s10649-006-9034-4

[B72] Östergren R. Träff U. (2013). Early number knowledge and cognitive ability affect early arithmetic ability. J. Exp. Child Psychol. 115, 405–421. 10.1016/j.jecp.2013.03.007 23665177

[B73] Passolunghi M. C. Lanfranchi S. (2012). Domain-specific and domain-general precursors of mathematical achievement: a longitudinal study from kindergarten to first grade. Br. J. Educ. Psychol. 82, 42–63. 10.1111/j.2044-8279.2011.02039.x 22429058

[B74] Peng P. Namkung J. Barnes M. Sun C. (2015). A meta-analysis of mathematics and working memory: moderating effects of working memory domain, type of mathematics skill, and sample characteristics. J. Educ. Psychol. 108, 455–473. 10.1037/edu0000079

[B75] Pickering S. Baqués J. Gathercole S. (1999). Batería de Tests de Memoria de Trabajo [Working Memory Tests Battery]. Barcelona: Universidad Autónoma de Barcelona.

[B76] Pintrich P. R. (2003). A motivational science perspective on the role of student motivation in learning and teaching contexts. J. Educ. Psychol. 95, 667–686. 10.1037/0022-0663.95.4.667

[B77] Pintrich P. R. Schunk D. H. (2002). Motivation in Education: Theory, Research and Applications. Columbus, OH: Merrill Prentice Hall.

[B78] Presentación M. J. Tena V. Mercader J. Colomer C. Siegenthaler R. Miranda A. (2015). Motivación y estilo atribucional sobre el rendimiento académico en Educación Infantil [Motivation and attributional style on academic achievement in Kindergarten]. Int. J. Dev. Educ. Psychol. 1, 101–110. 10.17060/ijodaep.2015.n1.v1.52

[B79] Reimann G. Stoecklin M. Lavallee K. Gut J. Frischknecht M. C. Grob A. (2013). Cognitive and motivational profile shape predicts mathematical skills over and above profile level. Psychol. Sch. 50, 37–56. 10.1002/pits.21659

[B80] Röthlisberger M. Neuenschwander R. Cimeli P. Roebers C. M. (2013). Executive functions in 5-to 8-year olds: developmental changes and relationship to academic achievement. J. Educ. Dev. Psychol. 3, 153–167. 10.5539/jedp.v3n2p153

[B81] Sarabia A. Iriarte C. (2011). El Aprendizaje de las Matemáticas? Qué Actitudes, Creencias y Emociones Despierta Esta Materia en los Alumnos? [The Learning of Mathematics. What Attitudes, Beliefs and Emotions Awake This Subject in Students?] Navarra: Eunse.

[B82] Sarama J. Clements D. H. (2009). Early Childhood Mathematics Education Research: Learning Trajectories for Young Children. Oxford: Routledge.

[B83] Sasanguie D. Göbel S. Moll K. Smets K. Reynvoet B. (2013). Acuity of the approximate number sense, symbolic number comparison or mapping numbers onto space: what underlies mathematics achievement? J. Exp. Child Psychol. 114, 418–431. 10.1016/j.jecp.2012.10.012 23270796

[B84] Satorra A. Bentler P. M. (1994). Corrections to tests statistics and standard errors in covariance structure analysis, in Latent Variables Analysis: Applications for Developmental Research, eds Von Eye A. Clogg C. C. (Thousand Oaks, CA: Sage), 399–419.

[B85] Sobel M. E. (1982). Asymptotic confidence intervals for indirect effects in structural equation models, in Sociological Methodology, ed Leinhardt S. (Washington, DC: American Sociological Association), 290–312. 10.2307/270723

[B86] Spreen O. Strauss E. (1991). A Compendium of Neuropsychological Tests: Administration, Norms, and Commentary. New York, NY: Oxford University Press.

[B87] Stock P. Desoete A. Roeyers H. (2007). Early markers for arithmetic difficulties. Educ. Child Psychol. 24, 28–39.

[B88] Stock P. Desoete A. Roeyers H. (2010). Detecting children with arithmetic disabilities from kindergarten: evidence from a 3-year longitudinal study on the role of preparatory arithmetic abilities. J. Learn. Disabil. 4, 250–268. 10.1177/0022219409345011 19903867

[B89] Tobia V. Bonifacci P. Marzocchi G. M. (2016). Concurrent and longitudinal predictors of calculation skills in preschoolers. Eur. J. Psychol. Educ. 31, 155–174. 10.1007/s10212-015-0260-y

[B90] Toll S. W. Van der Ven S. H. Kroesbergen E. H. Van Luit J. E. (2011). Executive functions as predictors of math learning disabilities. J. Learn. Disabil. 44, 521–532. 10.1177/0022219410387302 21177978

[B91] Turner J. C. Meyer D. K. (2009). Understanding motivation in mathematics: what is happening in classrooms?, in Handbook of Motivation at School, eds Wentzler K. R. Wigfield A. (New York, NY: Routledge), 527–552.

[B92] van der Sluis S. de Jong P. F. van der Leij A. (2004). Inhibition and shifting in children with learning deficits in arithmetic and reading. J. Exp. Child Psychol. 87, 239–266. 10.1016/j.jecp.2003.12.002 14972600

[B93] Veenman M. V. J. (2006). The role of intellectual and metacognitive skills in math problem solving, in Metacognition in Mathematics Education, eds Desoette A. Veenman M. (New York, NY: Nova Science Publishers), 35–50.

[B94] von Aster M. G. Shalev R. (2007). Number development and developmental dyscalculia. Dev. Med. Child Neurol. 49, 868–873. 10.1111/j.1469-8749.2007.00868.x 17979867

[B95] Wang A. H. Shen F. Byrnes J. P. (2013). Does the Opportunity–Propensity Framework predict the early mathematics skills of low-income pre-kindergarten children? Contemp. Educ. Psychol. 38, 259–270. 10.1016/j.cedpsych.2013.04.004

[B96] Wechsler D. (1996). Escala de Inteligencia de Wechsler para Preescolar y Primaria [Wechsler Intelligence Scale for Preschool and Primary]. Madrid: TEA Ediciones.

[B97] Weiner B. (1986). An Attributional Theory of Motivation and Emotion. New York, NY: Springer Science & Business Media.

[B98] Wigfield A. Eccles J. S. (2002). The development of competence beliefs, expectancies for success, and achievement values from childhood through adolescence, in Development of Achievement Motivation, eds Wigfield A. Eccles J. S. (San Diego, CA: Academic Press), 92–120. 10.1016/B978-012750053-9/50006-1

[B99] Wilson A. J. Dehaene S. (2007). Number sense and developmental dyscalculia, in Human Behavior, Learning, and the Developing Brain: Atypical Development, eds Coch D. Dawson G. Fischer K. W. (New York, NY: Guilford Press), 212–238.

